# Transcatheter arterial chemoembolization combined with simultaneous DynaCT-guided radiofrequency ablation in the treatment of solitary large hepatocellular carcinoma

**DOI:** 10.1007/s11547-018-0932-1

**Published:** 2018-08-21

**Authors:** Hongjun Yuan, Fengyong Liu, Xin Li, Yang Guan, Maoqiang Wang

**Affiliations:** Department of Interventional Radiology, General Hospital of People’s Liberation Army, 28 Fuxing Road, Beijing, 100853 People’s Republic of China

**Keywords:** Hepatocellular carcinoma, Therapeutic embolization, Interventional radiology, DynaCT

## Abstract

**Purpose:**

To introduce the technique and investigate the clinical efficacy of transcatheter arterial chemoembolization (TACE) in combination with simultaneous DynaCT-guided radiofrequency ablation (RFA) in the treatment of solitary large hepatocellular carcinomas (HCCs) (maximal diameter > 5 cm).

**Materials and methods:**

Forty-six patients who received TACE combined with simultaneous DynaCT-guided RFA for solitary large HCCs between January 2012 and August 2016 were reviewed, and the success rate, safety, local tumor progression (LTP), and overall survival (OS) were retrospectively investigated. OS and time to progression were analyzed with the Kaplan–Meier method.

**Results:**

Technical success rate was 100%, average operative time for DynaCT-guided RFA was 45.3 ± 4.8 min, average radiation dose was 730.5 ± 78.8 mGy, and no life-threatening complications were observed. At 1-month follow-up enhanced MRI, complete remission was achieved in 82.6% of patients (38/46), and partial remission in 17.4% (8/46). The median follow-up period was 29.5 months (interquartile range 4.0–69.0 months). At 1, 2, and 3 years after surgery, the LTP rates were 4.3, 13.1, and 30.4%, respectively, and the OS rates were 89.1, 71.7, and 56.5%, respectively.

**Conclusion:**

DynaCT-guided TACE + RFA is safe and feasible for the treatment of solitary large HCCS. TACE combined with simultaneous RFA provides a new treatment option for solitary large HCCs in which DynaCT has important clinical value.

## Introduction

Compared with other countries, large HCCs (maximal diameter > 5 cm) have a higher incidence in Asia, especially in China. This is primarily due to the large Chinese population, poor economic conditions, and limited awareness of proper treatment. Large HCCs are often identified at initial diagnosis [1]. The space-occupying effect of large HCCs may cause poor liver functional reserve and often compresses or invades the surrounding tissues which increase the risk of surgical intervention [2, 3].

For unresectable large HCCs, especially solitary large HCCs, transcatheter arterial chemoembolization (TACE) has become the primary treatments [4–6]. Of note, any single treatment has its own limitations and, thus, its efficacy is usually dissatisfactory. Currently, some clinicians recommend TACE combined with RFA or microwave ablation for the treatment of large HCCs [7]. However, sequential combined therapy is frequently employed, and ablation is usually performed 2–4 weeks after TACE [8]. Recently, researchers have treated HCCs using immediate combination with TACE and RFA under the guidance of cone-beam CT (CBCT) and satisfactory outcomes were achieved. Newer technologies such as the flat-panel detector digital subtraction angiography (DSA) system with CBCT have become popular in clinical practice, and perspective, photography, DSA, and 3D reconstruction/imaging can be performed simultaneously on the same working bed using DynaCT [such as the Artis Zee DSA system with CBCT (SIEMENS, Germany)] [9]. After TACE under DSA, DynaCT can be used for scanning and imaging and the puncture site and the route can then be determined. Thus, DynaCT can be used to guide puncture with the aid of the DSA system [10]. The use of DynaCT not only realizes simultaneous combination of TACE and RFA, but also maximizes the synergistic effect of TACE and RFA, increasing therapeutic efficacy [11]. The clinical value of TACE combined with simultaneous RFA has been gradually accepted by some clinicians. Ultrasound also could be simultaneously used to guide ablation after TACE [12], and that contrast-enhanced ultrasound and fusion imaging were another advanced technique for difficult cases [13]. The current study hypothesis was that DynaCT-guided TACE + RFA provides a new treatment option for solitary large HCCs. This study was undertaken to investigate the clinical value and safety of DynaCT in the treatment of solitary large HCCs.

## Materials and methods

### General patient characteristics

All patients were diagnosed with HCC by imaging or pathological examination and received TACE with simultaneous DynaCT-guided RFA. A total of 46 patients received treatment for solitary large HCCs in our hospital between January 2012 and August 2016. There were 32 males and 14 females (average age 56.34 ± 7.92 years; range 29–78 years) (Table 1).

**Table 1:** General characteristics of patients

Variables	Values
Gender (M/F)	32/14
Age/range (years)	56.34 ± 7.92/(29–78)
HBsAg	
Positive	42
Negative	4
Serum AST (IU/L)	44.27 ± 13.37 (22–88)
Serum ALT (IU/L)	39.15 ± 12.92 (19–72)
AFP (ng/mL)	
≥ 20	38
< 20	8
Child–Pugh grade	
A	18
B	28
Maximal tumor diameter/range (cm)	6.78 ± 0.87/(5.3–9.4)
Hepatic cirrhosis	
Yes	40
No	6
Cancer location	
Near subdiaphragmatic area	8
Non-subdiaphragmatic area	38

*AFP* alpha fetoprotein, *ALT* alanine aminotransferase, *AST* aspartate aminotransferase, *HBsAg* hepatitis B surface antigen

The inclusion criteria were as follows: (1) Imaging or pathological examination confirmed unresectable solitary HCCs and the maximal tumor diameter was > 5 cm; (2) the tumor selected for ablation was ≥ 1 cm away from the important organs and tissues (gallbladder, intestine, bile duct, and major vessels); (3) the Child–Pugh grade of liver function was A and B or BCLC grade was A and B, and the Karnofsky score was  >  70; and (4) patients received initial treatment.

The exclusion criteria were as follows: (1) patients were unsuitable for interventional treatment due to other serious diseases (such as coagulation disorder, prothrombin activity < 40%, platelet count < 30 × 109/L); (2) patients were sensitive to iodine and femoral puncture was infeasible; (3) patients had large arteriovenous fistula in the liver, involvement of the bile duct, tumor thrombus within the portal vein, and extrahepatic metastasis; (4) the expected survival time was shorter than 3 months; and (5) patients had poor lung function and the duration of breath holding was shorter than 8 s even after breathless training.

### Equipment

Artis zee DSA (Artis zee BA Twin; Siemens AG, Germany), Syngo Workplace workstation (Syngo X-workplace with Syngo DynaCT; Siemens AG, Germany), and radiofrequency therapeutic apparatus (Model 1500: RITA Medical System, Mountain View, CA, USA) were used in this study. A radio frequency of 460 kHz was chosen, and a RITA multipolar radiofrequency ablation electrode needle (outer tube diameter: 14G) with nine hook-shaped bundle electrodes (umbrella-like opening with the diameter of 5 cm) was used.

### Treatment methods

#### TACE treatment

After routine skin sterilization, local anesthesia with lidocaine was administered. Modified Seldinger catheter was used for femoral puncture, and a Simon I 4F catheter was employed for celiac artery and superior mesenteric artery angiography. If necessary, selective hepatic artery angiography was performed.

**Fig. 1:**
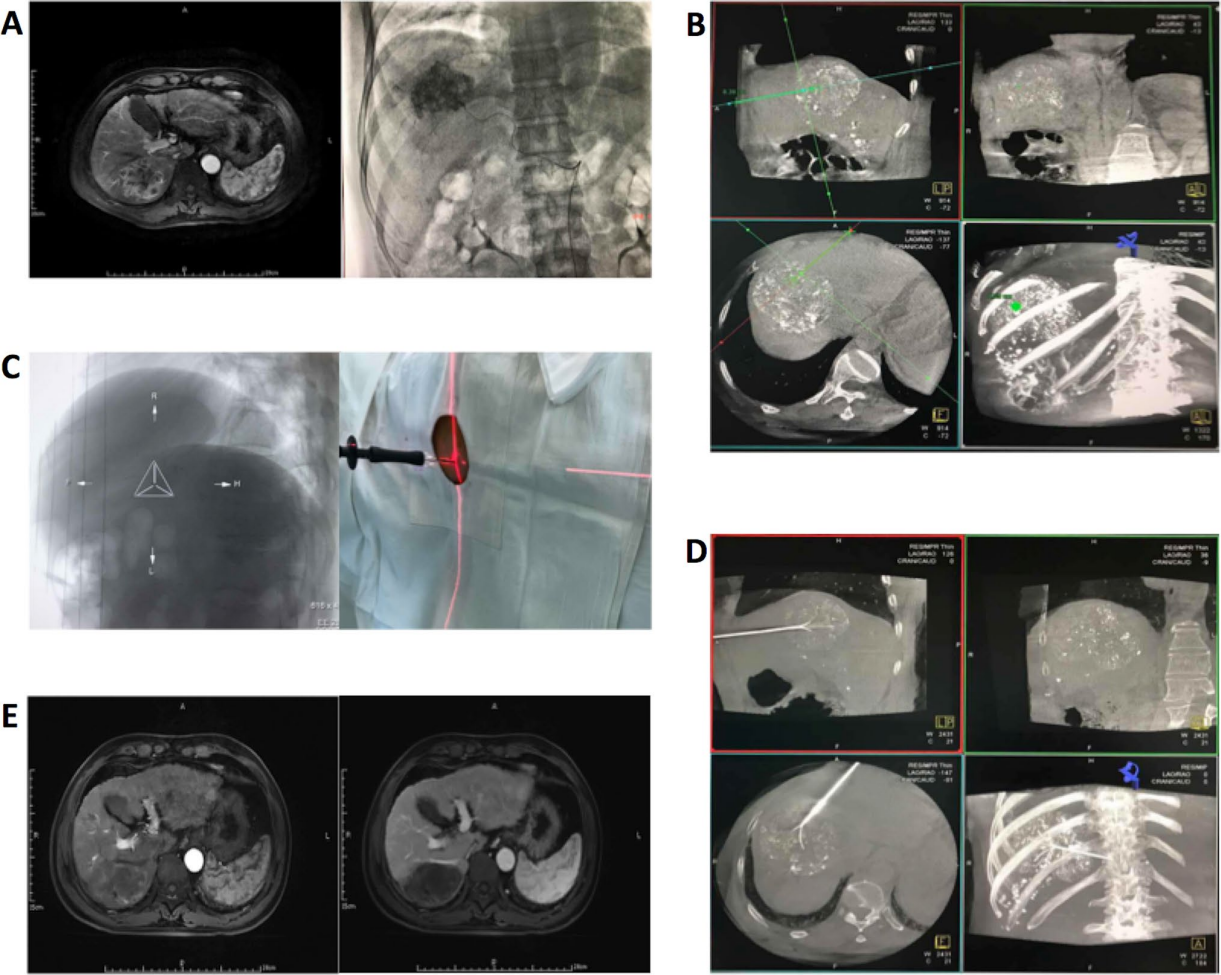
A59-year-old male. **a** Preoperative MRI shows a hypointense liver lesion and lipiodol embolism during TACE. **b** DynaCT performed immediately after TACE. The target site, surface site, and puncture routine are determined using the workstation. **c** Bull’s eye position; after laser localization, puncture is performed along the direction of laser. **d** After successful puncture, DynaCT is performed again to confirm the needle location. **e** MRI of the liver in the arterial phase and venous phase at 1 month after surgery

The artery supplying the tumor was determined by DSA. Then, a 2.6 F microcatheter was used for superselective catheterization of the supplying artery, followed by chemoembolization (Fig. 1a). The endpoint of TACE was stasis of feeding arterial flow. RFA was performed immediately in the region where iodine oil deposition is poor under the guidance of DynaCT.

### DynaCT-guided Syngo iGuide Needle puncture

#### DynaCT image acquisition

A DR scanning interval of 8 s was used for DynaCT, and the angle of the C-arm and location of the treatment bed were adjusted for the calibration of anterior–posterior and lateral X-rays. DynaCT of the abdomen was performed to include the area of lesion. The following parameters were used: rotation angle of C-arm: 200°; rate of image capture: 50 frames/s; increment in each frame: 0.5°; X-ray dose: 0.36 μGy/frame; total time: 8 s; and total number of frames: 396. Patients were asked to hold their breath during image acquisition, and the images were sent to the Syngo Workplace workstation for reconstruction of the three-dimensional volumetric images as well as axial, sagittal, and coronal images.

#### iGuide three-dimensional puncture and positioning

(1) Using the DynaCT workstation interface, the width and location of the windows were adjusted to clearly display the lesion. iGuide mode was selected, and a cross was used to localize the lesion in axial, sagittal, and coronal planes. The inner puncture target spot was selected, and the surface puncture was marked, according to the puncture routine automatically selected by the iGuide software (Fig. 1b); the depth of puncture was also displayed; (2) the bull’s eye position was selected, the operating lever was pushed, the C-arm was automatically fixed at the bull’s eye position, and then the inner puncture site was assured to overlap the surface puncture site. The laser positioning lamp was turned on, the laser cross focused on the tail of RF needle, and then the puncture location was adjusted (Fig. 1c). The operating lever was pushed to switch the C-arm at three different angles to guide the RFA. The puncture was performed along the pre-designed routine under the guidance of DynaCT; (3) once the needle tip reached the target site, DynaCT was performed again to confirm whether the puncture was successful (Fig. 1d).

#### RFA treatment

After puncture with the RF needle, the needle was opened to become multipolar umbrella-like. Once the needle tip was localized at the target, the 1500 RITA system was turned on for RFA. RFA was performed using a power of 150–200 W and temperature of 105 °C for 15–20 min. For the large tumor size, the needle location was adjusted after ablation at a site for further ablation until the ablated area covered the edge of the tumor and its three-dimensional space. Of note, DynaCT was performed again before the next ablation, and the routine was redesigned. After ablation, the needle was withdrawn, and the tunnel was ablated at 70–90 °C to reduce tunnel hemorrhage and implantation metastasis of the tumor along the tunnel. The puncture was compressed for 2–5 min, and a pressure dressing was administered with aseptic dressings.

### Follow-up

At 1 month after surgery, abdominal enhanced MRI of the abdomen was performed and the severity and extent of lesion necrosis and local recurrence were recorded (Fig. 1e). Any postoperative complications were promptly managed, and the survival status was monitored during follow-up. If residual cancer was present, a second TACE + RFA or RFA alone was performed, depending on the status of the residual cancer. If there was no residual cancer, re-examination was performed once every 3 months. The survival time was the interval from the first TACE operation to death. We studied the time to progression (TTP) and 1-, 2-, and 3-year survival rates.

### Response assessment

Technical success was defined as completion of both TACE and RFA in one treatment session. Initial tumor response was assessed by contrast-enhanced MRI 1 month after treatment according to the modified response evaluation criteria in solid tumor (m-RECIST) developed by the American Association for the Study of Liver Diseases (AASLD) [14]: Complete remission (CR), partial remission (PR), stable disease (SD), progressive disease (PD). Efficacy equaled CR plus PR. LTP was defined as the appearance of enhancement around or within the ablation zone that occurred at least 1 month after treatment and evaluated based on enhanced MRI.

### Statistical analysis

Statistical analysis was performed using the SPSS version 24.0 (SPSS Inc., Chicago, IL, USA). Quantitative data were expressed as mean ± standard deviation (*x* ± *s*). OS rates and LTP rates were estimated using the Kaplan–Meier method. A *P* value < 0.05(two-tailed) was considered statistically significant.

## Results

### Technical success rate, operative time, and radiation dose

Forty-six patients underwent TACE and simultaneous DynaCT-guided RFA. DynaCT-guided TACE and simultaneous RFA were performed by an experienced clinician in the Department of Radiology. For a lesion close to the important organs (peripheral lesion, subdiaphragmatic lesion, and lesion close to the intestine), lidocaine in normal saline was injected to separate the lesion from surrounding tissues, followed by puncture. The technical success rate was 100%. The average duration of DynaCT-guided RFA was 45.3 ± 4.8 min, and the average radiation dose was 730.5 ± 78.8 mGy (Table 2).

**Table 2:** Operative time and radiation dose

Index	Average operative time (min)	Average radiation dose (mGy)
DynaCT-guided TACE	50.4 ± 4.7 (23.5–80.7)	874.3 ± 131.3 (216.3–2428)
DynaCT-guided RFA	45.3 ± 4.8 (35–65)	730.5 ± 78.8 (510.4–1187)

*RFA* radiofrequency ablation, *TACE* transcatheter arterial chemoembolization

### Tumor response assessment and long-term survival

**Fig. 2:**
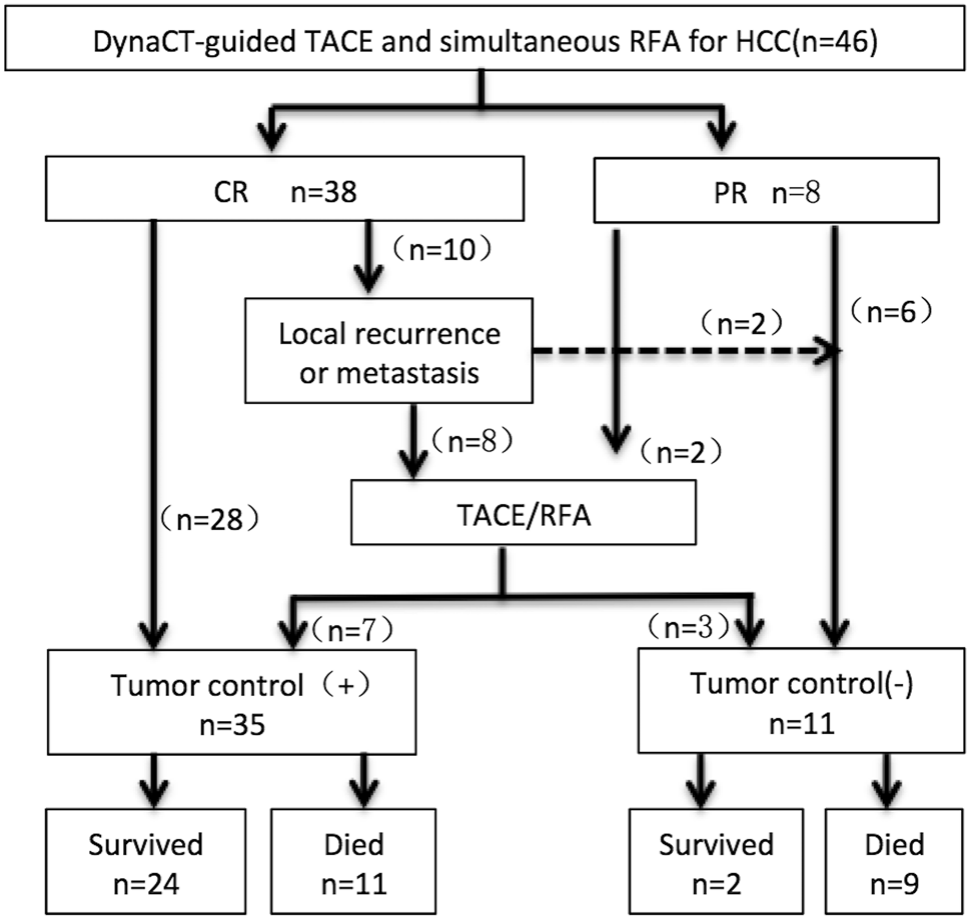
Therapeutic efficacy and survival status of 46 patients receiving TACE with simultaneous RFA

**Fig. 3:**
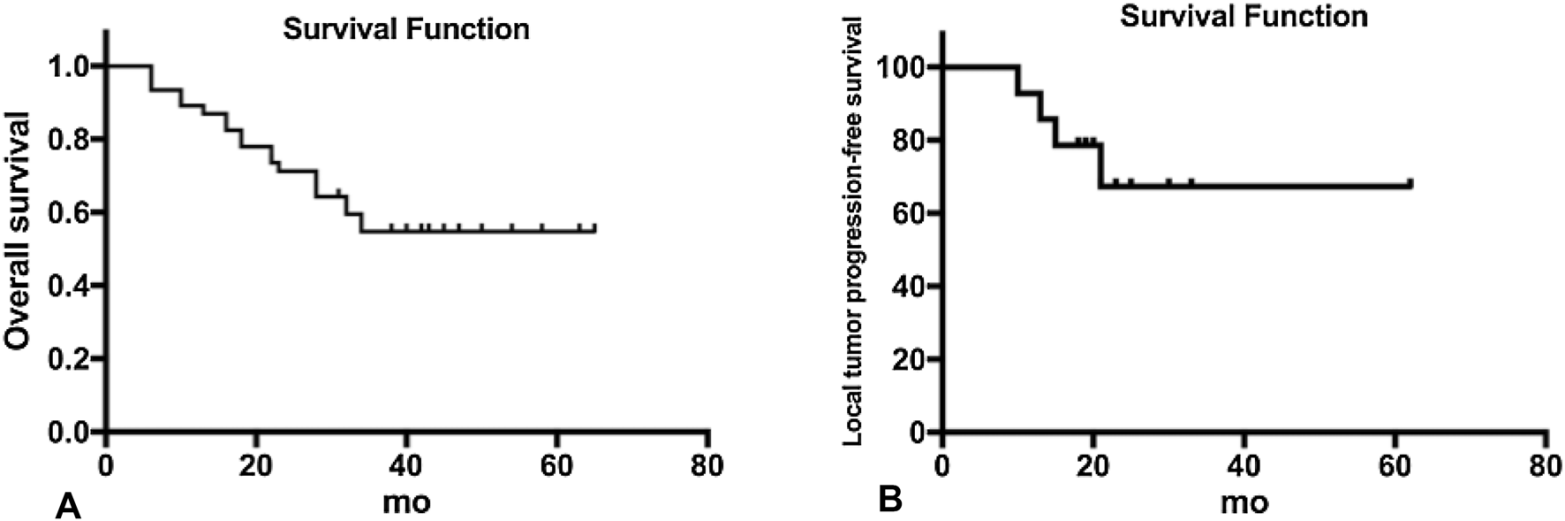
Overall survival after DynaCT-guided TACE with simultaneous RFA (**a**). Local tumor progression-free survival after DynaCT-guided TACE with simultaneous RFA (**b**)

Forty-six patients were followed up from the initial treatment to October 2017. The average duration of follow-up was 29.8 ± 17.3 months (range 4–69 months). At 1 month after surgery, the enhanced MRI was performed, and the therapeutic efficacy was assessed using m-RECIST criteria. The results showed a CR in 82.6% (38/46) of patients, and a PR in 17.4% (8/46) with a clinical efficacy rate of 100%. After initial treatment, ten patients received a second TACE or RFA during follow-up, of whom CR was achieved in seven patients (as the remaining patients did not receive further TACE or RFA due to tumor progression or personal rejection). During the follow-up period, the LTP was observed in 25 of 46 patients (54.3%). LTP was found in 14 patients (30.4%). At 1, 2, and 3 years after treatment, the LTP rates were 4.3% (2/46), 13.1% (6/46), and 30.4% (14/46), respectively. During the follow-up period, 17 patients died from tumor progression, two died from gastrointestinal bleeding, and one died from hepatic encephalopathy. One, 2, and 3 years OS rates were 89.1% (41/46), 71.7% (33/46), and 56.5% (26/46), respectively (Figs. 2, 3).

### Intraoperative and postoperative complications

Complications were evaluated according to the Cardiovascular and Interventional Radiological Society of Europe (CIRSE) classification system [15]. No grades 6, 5, and 4 complication was related to procedures. The occurrence rate of grade 1 was 100%, such as discomfort or pain over the hepatic region of varying extent and transient abnormalities in routine blood. The occurrence rate of grade 2 was 82.6%, included 38 patients had fever (body temperature: 37.5–39.8 °C) and 21 patients developed microscopic hematuria. The occurrence rate of grade 3 was 6.5%, included subcapsular hematoma of liver (*n* = 2), sand diaphragmatic damage with pleural effusion (*n* = 1).

## Discussion

In recent years, RFA has become a common nonsurgical treatment besides TACE and its effectiveness has been confirmed in small HCCs, comparable to that of radical treatments (such as surgical intervention and liver transplantation). However, RFA cannot assure peripheral necrosis for large or irregular HCCs, which increases the risk for residual tumor, recurrence, and metastasis [16]. With the development of RFA instrumentation and imaging guidance equipment and the accumulation of clinical experience, RFA has been increasingly used in the treatment of large HCCs [17]. In particular, the introduction of CBCT has realized the combination of TACE with simultaneous RFA, providing a new, convenient, and effective treatment for large HCCs. Morimoto et al. [18] reported the treatment of HCCs with CBCT-guided RFA. In their study, the RF needle accurately punctured the target lesion in six patients, but the puncture was affected by body position or respiration in two patients. Wang et al. [14, 19] employed TACE with simultaneous CBCT-guided RFA in the treatment of large HCCs in 21 patients, with a technical success rate of 100%.

DynaCT employs CTCB using the Artis zee DSA system (SIMENS, Germany) and can realize synchronized acquisition with flat-panel detector during C-arm rotation. After image reconstruction using the workstation, three-dimensional images of the target lesion and CT images of soft tissues can be obtained, which overcomes the limitations of traditional instruments. Thus, spiral CT and DSA can be performed on the same treatment bed. Using this technique, DynaCT-guided RFA was employed for the treatment of HCCs in this study. In the present study, DynaCT-guided RFA was conducted immediately after DSA-guided TACE with a technical success rate of 100%. At 1, 2, and 3 years after surgery, the survival rate was 89.1% (41/46), 71.7% (33/46), and 56.5% (26/46), respectively, which is consistent with the survival rates reported by Takaki et al. [20]. These results demonstrate favorable short-term efficacy of this treatment. In 46 patients, no intraoperative and postoperative severe complications were observed, suggesting favorable safety of this technique.

Based on our findings, DynaCT-guided TACE with simultaneous RFA appears safe and reliable for treatment of solitary large HCCs. The advantages of this technique include simultaneous combination of TACE and RFA which reduces the time interval between two treatments, thus avoiding the clearance of lipiodol and chemotherapeutics and the formation of new collateral vessels and vascular recanalization after embolization in this time interval [21]. In addition, DynaCT-guided TACE with simultaneous RFA may exert a synergistic effect, i.e., RFA after complete lipiodol deposition may maximize the heat conduction effect of the lipiodol, and, therefore, the heat produced by the RF needle can focus on the site of lipiodol deposition, exerting maximum anti-tumor effect; this is important for irregular large HCCs because peripheral tumor is difficult to completely ablate by RFA, but RFA after complete lipiodol deposition improves transduction of heat to peripheral tissues, which reduces the risk for recurrence and metastasis [19, 22]. With rotation of the C-arm, the patients can receive RFA on the same treatment bed without movement after TACE, which avoids the transfer of patients which normally occurs during traditional interventional therapy. This simplifies the procedures, reduces the risk of surgery, and improves therapeutic safety. Finally, the instrument can provide information in a real-time manner, which is helpful for the adjustment of treatment protocol; DynaCT scanning after TACE can accurately display and identify lipiodol deposition in normal liver tissues, which is helpful for decision making in surgery (i.e., whether or not additional lipiodol injection is needed) [23].

Of note, DynaCT has several limitations including: (1) the puncture should be continuously monitored by DynaCT, which increases the radiation exposure to clinicians; (2) the images obtained from DynaCT should be subjected to MPR reconstruction in the workstation, and the reconstructed images have poor resolution and poor intensity differences between tissues as compared with those from routine CT [24]. Besides, the DynaCT-guided TACE with simultaneous RFA still had a limitation, which was the X-ray exposure. In several centers, US (or CEUS or fusion imaging) is used instead of DynaCT in order to reduce X-ray exposure. In this study, we tried our best to overcome these disadvantages and accumulated experience on this technique. The inclusion and exclusion criteria were strict, i.e., patients should receive complete preoperative examinations, the functional reserve of the liver should be carefully evaluated, liver-protective treatment should be administered after surgery to avoid liver failure, especially for those with poor liver reserve function, chemotherapeutics acting on cell division or proliferation were preferred, and two or more chemotherapeutics were recommended for chemotherapy [25]. The injection of lipiodol and embolizing agent were kept as even as possible to avoid reflux of lipiodol and ectopic embolization. High doses of lipiodol and chemotherapeutics were avoided, and the doses of lipiodol and chemotherapeutics and the time interval between two treatments were individualized. Before the DynaCT-guided puncture, clinicians instructed the patients to control their respiration to avoid motion artifacts due to respiration on the imaging. The length of the RF needle was of an appropriate length as a long RF needle may affect the rotation of the ball tube. In addition, local analgesic treatment with lidocaine was administered during surgery because patients receiving combined treatment experience longer operative times.

## Conclusions

Our results indicated that DynaCT-guided TACE with simultaneous RFA is a safe and efficacious treatment for solitary large HCCs. TACE combined with simultaneous RFA provides a new treatment option for solitary large HCCs in which DynaCT has important clinical value. When CT-guided interventional therapy is unavailable or the conditions of the surgical unit are less advanced, DynaCT is recommended for RFA or TACE with simultaneous RFA in the treatment of large HCCs with relatively complete capsules and favorable locations.
